# Socioeconomic Status, Rurality, and Pediatric Critical Care Admission

**DOI:** 10.1001/jamanetworkopen.2026.3594

**Published:** 2026-03-26

**Authors:** Jeffrey N. Bone, Ye Shen, Stella Harden, Jennifer Retallack, Matthew Carwana, Srinivas Murthy, Fiona Muttalib

**Affiliations:** 1 BC Children’s Hospital Research Institute, Vancouver, Canada; 2 Department of Statistics, University of British Columbia, Vancouver, Canada; 3 Department of Geography, Simon Fraser University, Burnaby, Canada; 4 Division of Critical Care Medicine, Department of Pediatrics, University of British Columbia, Vancouver, Canada; 5 Division of Pediatric Hospital Medicine, Department of Pediatrics, University of British Columbia, Vancouver, Canada

## Abstract

**Importance:**

Neighborhood-level socioeconomic deprivation has been associated with higher incidence and severity of pediatric critical illness; however, structural factors underlying observed differences have received limited attention.

**Objective:**

To describe the association between area-level deprivation, rurality, and incidence of critical care admission among children.

**Design, Setting, and Participants:**

This retrospective cohort study used linked data of individuals aged 0 to 17 years who were admitted to intensive care units in British Columbia (BC), Canada, between 2014 and 2023. Data were analyzed from June to November 2025.

**Exposures:**

Age, sex, rurality, and neighborhood situational vulnerability (a measure of socioeconomic disadvantage).

**Main Outcomes and Measures:**

The primary outcome was the incidence rate of critical care admission. Poisson regression models were used to estimate incidence rate ratios (IRRs) between exposure groups. The possible interaction between situational vulnerability quintile and population center type was examined.

**Results:**

A total of 10 048 children were admitted 13 990 times to intensive care units (incidence rate of 154 per 100 000 person years). Most admissions were for male children (7641 [54.6%]) and children younger than 5 years (7528 [53.8%]). The IRR for rural or small population centers compared with medium or large was 1.35 (95% CI, 1.28-1.41). Similarly, the IRR for the most vs least deprived quintile was 1.31 (95% CI, 1.23-1.39); however, in medium or large population centers, there was no association between situational vulnerability quintile and critical care admission (eg, quintile 5 vs 1: IRR 1.04; 95% CI, 0.97-1.12). Those in rural or small areas of the most deprived quintile had the highest overall rate (IRR, 2.02; 95% CI, 1.87-2.19 vs highest quintile in large or medium population centers).

**Conclusions and Relevance:**

In this retrospective cohort study there was higher burden of critical care admission for children living in rural areas and small population centers, and areas with higher situational vulnerability. The association between situational vulnerability and critical care incidence was unique to rural or small regions. Targeted strategies are needed to address contributing factors and ensure timely access to pediatric acute care in underserved areas.

## Key Points

**Question:**

In an area with a universal health system, does rurality modify the association of neighborhood socioeconomic disadvantage with rate of pediatric critical care admission?

**Findings:**

In this cohort study of 13 990 pediatric critical care admissions, rates of critical care admission were higher in areas of greater socioeconomic disadvantage in rural and small areas but not in medium or large population centers.

**Meaning:**

These findings suggest that protective systemic and structural factors in medium and large population centers may mitigate neighborhood socioeconomic disadvantages.

## Teaser

This cohort study examines the association between area-level deprivation, rurality, and the incidence of critical care admission among children in British Columbia, Canada, between 2014 and 2023.

## Introduction

Neighborhood-level socioeconomic deprivation is associated with increased population-level incidence of pediatric critical illness and increased illness severity at the time of critical care admission.^1,2,3^ Neighborhood-level structural and systemic factors may affect the affordability and quality of housing, availability of education and employment opportunities, risk of hazardous environmental exposures, and accessibility of preventive health care and health-promoting behaviors. Among children who develop chronic health conditions or acute illnesses, timely access to preventive and acute care services is essential.

In Canada, as in many regionalized health care systems, specialized pediatric services are concentrated in urban areas.^4,5^ Due to Canada’s expansive geography, to reach specialized care, children living in rural areas and small population centers must be transported significant distances. When these children require interfacility transport to reach pediatric critical care services, they may have greater illness severity at admission and higher need for critical care interventions compared with children who present directly to the admitting hospital.^6,7,8^ In rural areas, challenges in access to health care services may compound the effects of neighborhood socioeconomic disadvantage.^9,10^ Little is known about the potential interaction between neighborhood level socioeconomic disadvantage and rurality on the incidence of pediatric critical illness.

The objective of this study was to characterize incidence rates of pediatric critical care admission in British Columbia (BC), Canada, and to describe the association and interaction between rurality, socioeconomic deprivation, and incidence of critical care admission.

## Methods

This study is reported according to the Strengthening the Reporting of Observational Studies in Epidemiology (STROBE) reporting guideline. Research ethics approval was obtained from the University of British Columbia. The requirement for participant informed consent was waived by the reviewing ethics board.

### Study Design and Setting

We conducted a population-level, retrospective cohort study of individuals younger than 18 years enrolled in the BC provincial government health services plan (Medical Services Plan [MSP]) between January 1, 2014, and December 31, 2023. Those not enrolled in MSP were excluded because the MSP registration file contains the key study exposure variables (age, sex, and place of residence, which allowed linkage to area-level variables).

**Figure 1.**
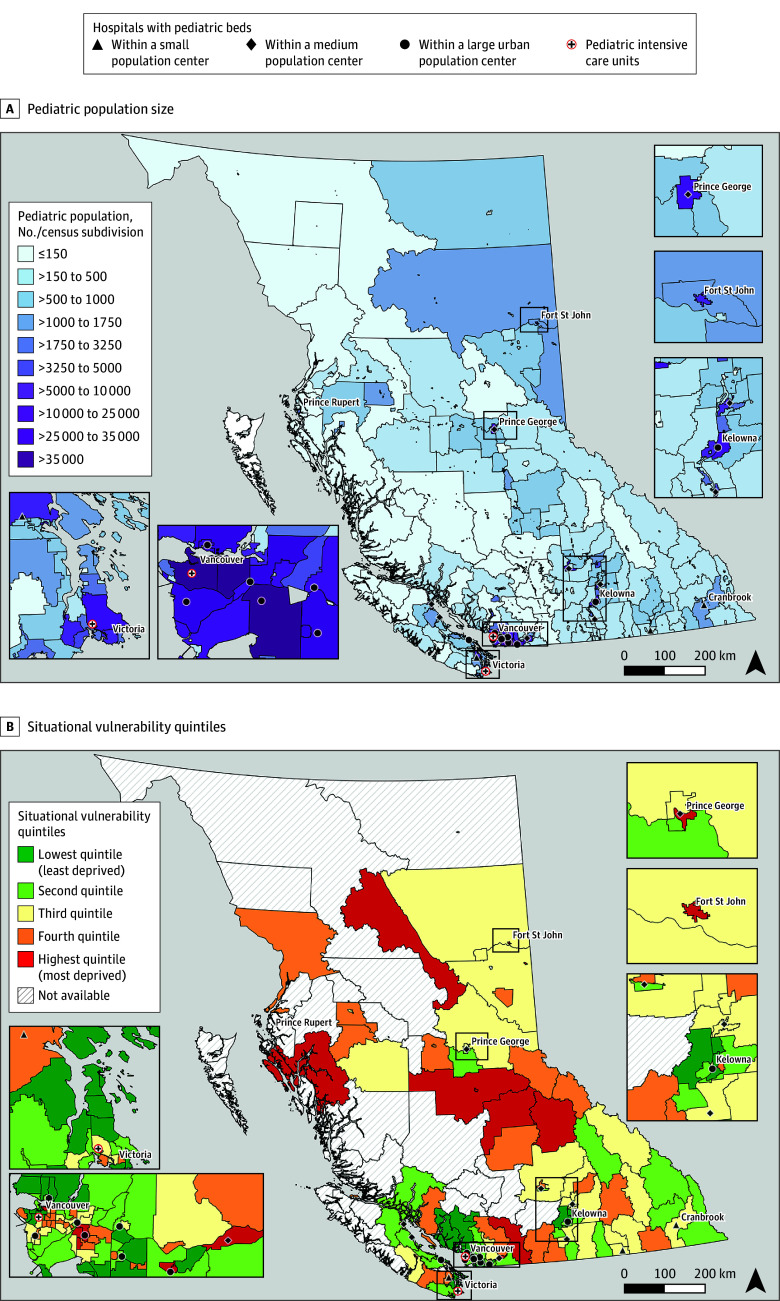
Map of British Columbia, Canada, Illustrating Hospitals With Pediatric Beds and Pediatric Population Size (Top) or Situational Vulnerability Quintile (Bottom)

In BC, there are 2 hospitals with pediatric intensive care units (PICUs) located in large population centers (Figure 1) and 30 hospitals with combined medical and surgical intensive care units (ICUs), primarily admitting adults. Children who require ongoing critical care are preferentially referred to the dedicated PICUs; however, due to geographic and logistical constraints, including extreme weather conditions, they may also be treated locally, outside the PICU setting (eg, adult ICUs).

### Data Sources

This study used linked retrospective data from Population Data BC. Access to data provided by the data stewards is subject to approval but can be requested for research projects through the data stewards or their designated service providers.

The following datasets were used in this study: Discharge Abstract Database (DAD), which captures all hospital admissions in BC, the Medical Services Plan consolidation file, which comprises all individuals registered to BC’s universal health care system, and Vital Events and Statistics Deaths, which captures all deaths in BC. Birth and mortality data were only accurate to the month and year and did not include exact date. Further information can be found in the Data Sharing Statement in Supplement 1.

### Participants

We defined an episode of pediatric critical illness as a unique hospital record in the DAD with special care unit (SCU) days in a PICU, combined medical or surgical ICU, coronary care unit, or cardiac ICU.^11,12^ Readmissions to special care units within the same hospital stay were counted as a single episode of pediatric critical illness. We included admissions to out-of-province ICUs where patients were transferred from a hospital in British Columbia. We excluded patients admitted only to neonatal ICUs (defined as SCU days in a neonatal ICU level 1, 2, or 3) and individuals admitted only to step-down medical and surgical units as these units do not admit critically ill children.

### Exposures and Variables of Interest

We assessed the following exposure variables for differences in incidence rates of critical care admissions: (1) age at time of admission, grouped according to Canadian Institute for Health Information (CIHI) categories (0 to 11 months, 1 to 4 years, 5 to 9 years, 10 to 14 years, and 15 to 17 years); (2) sex assigned at birth; (3) place of residence (population center type) and (4) neighborhood situational vulnerability, an area level measure of multiple deprivation. Place of residence was defined using the 6-digit residential postal code recorded in the MSP registry file for each exposure year. The Statistics Canada Population Centre and Rural Area Classification was used to define large (≥100 000 inhabitants), medium (30 000 to 99 999 inhabitants), and small (1000 to 29 999 inhabitants) population centers and rural areas (<1000 inhabitants). To describe socioeconomic status, we used quintiles of the BC Index of Multiple Deprivation situational vulnerability domain (BCIMD 2021), which is comprised of area-level information about the proportion of households without high school education, proportion of homes needing repair, proportion of single parent families, proportion of population self-employed, median household income, and home values.^13^

We used the primary *International Statistical Classification of Diseases and Related Health Problems, Tenth Revision* Canada diagnosis code to categorize diagnosis for the index hospital admission according to the Pediatric Clinical Classification System (PECCS-CA) and group admissions into (1) medical, (2) surgical, or (3) medical and surgical.^14^ We determined if patients were living with chronic medical conditions (CMC) with and without technology dependence using previously described methodology.^15^ Additional information about the indicators can be found in eMethods in Supplement 2.

### Outcome Measures

The primary outcome was the incidence rate of critical care admission. For each calendar year, incidence was defined as the number of children who were admitted to a critical care unit in BC divided by the number of eligible person years. We censored patients when they died, reached age 18 years, or unregistered from MSP (eg, due to migration). For population denominators, we used annual stratified population estimates based on the number of individuals younger than age 18 years registered to BC’s Medical Services Plan (MSP) (eMethods and eFigure 1 in Supplement 2). The following secondary outcomes were also summarized: length of hospital stay, total SCU days within the hospitalization, and mortality at 48 hours, in-hospital, and at 12 months from hospital admission.

### Statistical Analysis

We summarized the total number of critical care admissions and their characteristics using counts and frequencies for categorical variables and medians and IQRs for continuous variables. We calculated the incidence rate (per 100 000) by calendar year overall and by age, sex assigned at birth, population center size, and deprivation index quintile. We used Poisson regression models for annual counts to compute incidence rate ratios (IRR) and incidence rate differences (IRD; per 100 000).^16^ Models included group by year interactions to allow for changes over time and offset terms for total person years of follow up. IRR and IRD confidence intervals were calculated using the model fitted values, averaged over the study period, with corresponding 95% delta-method CIs. In line with modern guidance for epidemiological studies, we based our interpretation of numerical results on the effect sizes and the values within the 95% CI, rather than on null hypothesis significance testing. For stratification by population center size and deprivation index quintile, we compared raw incidence rates to age- and sex-standardized incidence rates. Standardization was based on the annual age and sex-specific distribution of BC population. For sex, we examined age-specific IRRs between males and females. Gender data were unavailable; thus, gender-based analyses were not performed.

We examined possible effect modification of situational vulnerability quintile by population center size by inclusion of an interaction term in the above Poisson models. Due to small strata sizes, in primary interaction analyses we combined rural and small centers and compared with medium and large. Following best practice for reporting interactions we report measures on both multiplicative and additive scales.^13^ For multiplicative interaction, we estimated population center size specific IRRs for increased situational vulnerability quintile (vs highest quintile) and joint quintile-center associations (vs lowest deprivation in medium and large center). For additive interaction we estimated the relative excess risk due to interaction (RERI). The significance of the interaction term was tested by comparing models with and without interaction using the likelihood ratio test.

We conducted 2 sensitivity analyses. First, to determine whether results were influenced by a small number of patients with frequent readmissions, we conducted a sensitivity analysis including only 1 admission per patient. Second, we stratified the above results by urgent vs elective admissions.

Mortality incidence rates were calculated per 100 000 person years. Length of stay and total number of SCU days within the hospitalization were summarized descriptively. We examined trends in medical complexity over time and summarized the most common diagnosis overall and stratified by urgent vs elective admissions.

All variables had less than 1% missing data other than BCIMD quintile, which was suppressed for 4.5% of admissions (eMethods in Supplement 2). Complete case analyses were conducted. Analyses were conducted using R version 4.4.4 (R Project for Statistical Computing). Data were analyzed from June to November 2025.

## Results

### Admission Characteristics

A total of 10 048 children experienced 13 390 episodes of critical care between 2014 and 2023 for a total incidence rate of 154 admissions per 100 000 person years. Of the 10 048 patients admitted, 8826 (85.0%) were admitted once, 929 (9.0%) were admitted twice, and 653 (6.0%) were admitted 3 or more times.

Most patients admitted were younger than 5 years (7528 [53.8%]), male (7641 [54.6%]), and admitted only to a pediatric ICU (11 675 [83.5%]) or only to a combined medical surgical ICU (1944 [14.5%]). Children living in large population centers made up more than half (7918 [57.0%]) of admissions, while those from small and rural areas included 4520 admissions (32.6%). A total of 8431 (67.4%) and 4432 (31.7%) were urgent and elective admissions, respectively (Table 1).

**Table 1.** Demographic of Critical Care Admissions in British Columbia (BC), Canada, Between 2014 and 2023

Characteristic	No. of admissions (%)
No.	13 390
Demographic	
Age	
Median (IQR), years	4.0 (0-12.0)
0-11 mo	3901 (27.9)
1-4	3627 (25.9)
5-9	2212 (15.8)
10-14	2245 (16.0)
15-17	2005 (14.3)
Sex assigned at birth	
Female	6348 (45.4)
Male	7641 (54.6)
Population center type, No. inhabitants^a^	
Large (≥100 000)	7918 (57.0)
Medium (30 000 to 99 999)	1442 (10.4)
Small (1000 to 29 999)	2230 (16.1)
Rural (<1000)	2290 (16.5)
Situational vulnerability quintile^b^	
First (least)	2475 (17.7)
Second	2912 (20.8)
Third	3166 (22.6)
Fourth	2907 (20.8)
Fifth (most)	1934 (13.8)
Unknown	596 (4.3)
Admission	
Admission reason^c^	
Medical	7665 (55.1)
Surgical	2601 (18.7)
Medical and surgical	3635 (26.1)
Admission type	
Urgent	9431 (67.4)
Elective	4432 (31.7)
Newborn	127 (0.9)
Medical complexity	
None	6865 (49.1)
Single system with technology dependence	2204 (15.8)
Single system without technology dependence	1461 (10.4)
Multisystem with technology dependence	3012 (21.5)
Multisystem without technology dependence	448 (3.2)
Special care unit type	
PICU only	11 675 (83.5)
PICU with combined medical and surgical ICU	247 (1.8)
PICU with other SCU	11 (0.1)
Combined medical surgical ICU only	1944 (14.5)
Combined medical surgical ICU with other SCU	11 (0.1)
Coronary care unit	46 (0.3)
Cardiac ICU^d^	56 (0.4)
Admitted to from province hospital ICU	278 (2.0)
Length of hospital stay	
Median (IQR), d	5 (2-10)
≤ 1	2277 (16.3)
2-3	3331 (23.8)
4-6	3092 (22.1)
7-13	2817 (20.1)
14-27	1390 (9.9)
≥ 28	1083 (7.7)
No. of SCU days during hospitalization	
Median (IQR)	2 (1-3)
≤ 1	6977 (49.9)
2-3	3559 (25.4)
4-6	1642 (11.7)
7-13	1094 (7.8)
14-27	423 (3.0)
≥ 28	295 (2.1)
Mortality	
12-mo mortality^e^	555 (5.5)^f^
During index hospital stay	369 (66.5)^g^
Within 48 h of admission	152 (27.4)^g^
Within 12 mo postdischarge	186 (33.5)^g^

Abbreviations: ICU, intensive care unit; PICU, pediatric intensive care unit; SCU, special care unit.

^a^ Based on Statistics Canada.

^b^ Based on BC Index of Multiple Deprivation income data.

^c^ Characterized using the Pediatric Clinical Classification System-Canada classification.

^d^ Adult ICUs as BC does not have a dedicated pediatric cardiac ICU.

^e^ Date of death only available to within 1 month.

^f^ Denominator is total number of children admitted (10 048 children).

^g^ Denominator is for total deaths.

The most common reasons for critical care admission overall were respiratory, with acute bronchiolitis (816 [5.8%]), pneumonia (628 [4.5%]), and asthma (452 [3.2%]) being the top 3 reasons for admissions (eTable 1 and 2 in Supplement 2). Additionally, 6865 admissions (49.1%) were for patients with single system medical complexity and 3460 (24.7%) of admissions were for children with multisystem medical complexity (Table 1). There was minimal change in prevalence of CMC among critical care admissions over the study period (eFigure 2 in Supplement 2). A total of 555 children (5.5%) died either in the hospital or within 12 months of hospital discharge (incidence rate, 6.4 per 100 000 person years); 369 (66.5%) died during the index hospitalization, and 186 (33.5%) died postdischarge (Table 1).

### Incidence Rates by Groups

The incidence rate of ICU admission was highest among those younger than 1 year, declined up to 10 to 14 years, followed by a small increase in those aged 15 to 17 years. Incidence rates were higher among male vs female children (IRR, 1.14; 95% CI, 1.10-1.18) (Table 2), due to differences in those younger than 4 years (eTable 4 in Supplement 2).

**Table 2.** Results by Age Group, Sex, Rurality, and Income Quintile

Characteristic	IR (per 100 000 person years)	IRR (95% CI)	IRD (95% CI) per 100 000 person years
Age group, y			
0-11 mos	949.0	1 [Reference]	1 [Reference]
1-4	199.3	0.21 (0.20 to 0.22)	−743 (−773 to −712)
5-9	90.6	0.10 (0.09 to 0.10)	−851 (−881 to −821)
10-14	90.6	0.10 (0.09 to 0.10)	−851 (−881 to −821)
15-17	129.2	0.14 (0.13 to 0.14)	−812 (−843 to −782)
Sex			
Female	150.1	1 [Reference]	1 [Reference]
Male	170.9	1.14 (1.10 to 1.18)	21 (16 to 27)
Population center type, No. inhabitants			
Large (≥100 000)	146.4	1 [Reference]	1 [Reference]
Medium (30 000 to 99 999)	147.4	1.00 (0.94 to 1.05)	−1 (−9 to 8)
Small (1000 to 29 999)	210.6	1.44 (1.37 to 1.51)	64 (55 to 73)
Rural (<1000)	199.9	1.35 (1.28 to 1.41)	51 (42 to 59)
Situational vulnerability, quintile			
First (least deprived)	144.4	1 [Reference]	1 [Reference]
Second	143.0	0.99 (0.94 to 1.05)	−1 (−9 to 6)
Third	169.7	1.18 (1.12 to 1.24)	25 (17 to 34)
Fourth	161.2	1.12 (1.06 to 1.18)	17 (9 to 25)
Fifth (most deprived)	189.6	1.31 (1.23 to 1.39)	45 (35 to 55)

Abbreviations: IRD, incidence rate difference; IRR, incidence rate ratio.

There was a higher incidence of critical care admission among children residing in rural or small population centers compared with medium or large population centers (IRR, 1.35; 95% CI, 1.28-1.41). Finally, there was a dose-response association between higher situational vulnerability quintile (ie, greater deprivation) and increased incidence of critical care admission. Quintiles 1 and 2 had similar incidence, while quintile 3 (IRR, 1.18; 95% CI, 1.12 to 1.24), quintile 4 (IRR, 1.12; 95% CI, 1.06 to 1.18), and quintile 5 (IRR, 1.31; 95% CI, 1.23 to 1.39) had moderately higher incidence rates (Table 2). The group specific incidence rates were largely unchanged after age and sex standardization (eTable 3 in Supplement 2).

### Effect Modification

**Figure 2.**
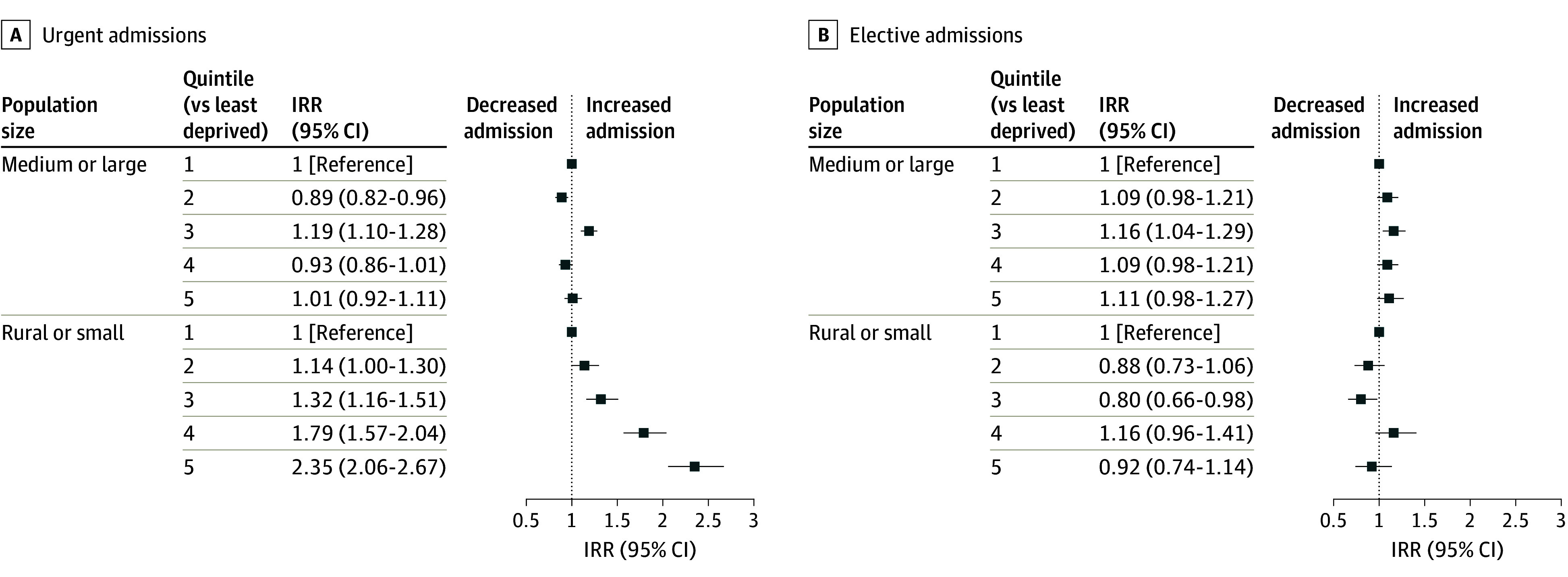
Forest Plot of Incidence Rate Ratios (IRRs) for Situational Vulnerability Quintile (vs Least Deprived) Within Strata of Small or Rural and Medium or Large Centers by Type of Admission Quintile 5 is most deprived.

The association between situational vulnerability quintile and incidence of critical care admission varied considerably by population center type. In medium and large population centers, there was no association between situational vulnerability quintile and incidence of critical care admission (eg, quintile 5 vs 1: IRR, 1.04; 95% CI, 0.97-1.12). In small population centers and rural areas there was a clear dose-response association between deprivation quintile and incidence of critical care admission (eg, quintile 5 vs 1: IRR, 1.83; 95% CI, 1.65-2.02) (eFigure 3 in Supplement 2). Those living in the most deprived quintile in small and rural centers had the highest risk overall and there was strong evidence of additive interaction for quintiles 4 and 5 based on the RERIs (Table 3). In sensitivity analysis, the association between situational vulnerability quintile and critical care admission in small and rural areas was only present for urgent admissions, and not for elective admissions (Figure 2 and eTable 5 in Supplement 2). Results were consistent when restricting to a single admission per patient (eTable 6 in Supplement 2).

**Table 3.** Joint Associations of Situational Vulnerability Quintile and Rurality (Small or Rural vs Medium or Large) With Incidence of Critical Care Admission^**a**^

Rurality	Incidence rate (per 100 000 person years)	Incidence rate ratio (95% CI)^b^	Relative excess risk due to interaction (95% CI)
Small or rural, situational vulnerability quintile			
1	157.5	1.10 (1.00 to 1.21)	NA
2	165.0	1.16 (1.07 to 1.25)	0.09 (−0.04 to 0.23)
3	178.0	1.25 (1.15 to 1.35)	−0.03 (−0.17 to 0.12)
4	246.5	1.73 (1.59 to 1.87)	0.64 (0.48 to 0.79)
5	289.7	2.02 (1.87 to 2.19)	0.88 (0.70 to 1.05)
Medium or large			
1	142.5	1 [Reference]	NA
2	136.4	0.95 (0.90 to 1.01)	NA
3	166.9	1.17 (1.10 to 1.24)	NA
4	140.3	0.99 (0.92 to 1.05)	NA
5	148.8	1.04 (0.97 to 1.12)	NA

Abbreviation: NA, not applicable.

^a^ *P* < .001 from likelihood ratio test comparing models with and without interaction between situational vulnerability and rurality.

^b^ Joint associations vs medium or large centers with lowest deprivation quintile.

## Discussion

In this population-based cohort study from BC, Canada, there was substantial variation in the incidence of pediatric critical care admission by place of residence. Incidence rates of urgent critical care admission were higher in areas of greater situational vulnerability in small and rural population centers but not in large and medium population centers. Children living in the most situationally deprived small and rural areas had the highest risk of critical care admission.

### Demographics in Context

Demographic characteristics of our study cohort are comparable to existing population-based reports of pediatric critical care in the US, the UK, Australia, and New Zealand.^14,15,17,18^ Population-based incidence rates of critical care admission in children have not yet been published in Canada. Incidence rates in our study cohort are similar to those reported in the UK and Ireland (145 per 100 000 child years in 2023) and lower than those reported in Australia and New Zealand (200 per 100 000 child years in 2021 and 2022).^14,17^ Differences in incidence rates of pediatric critical care admission may be explained by true population differences in incidence of critical illness as well as differences in health system structure, criteria for ICU admission, ICU bed availability, and methodology used for cohort definitions. The UK and Ireland have a comparable number of PICU beds per capita (2.7 per 100 000 children in the UK and Ireland vs 2.8 per 100 000 children in British Columbia) while Australia and New Zealand reported 3.8 beds per 100 000 children in 2019.^6,14,17^

### Place of Residence

Areas of greater socioeconomic disadvantage have been associated with higher area-level incidence of PICU admission in the UK, the US, and Australia.^1,2,3,19^ In Australia, in areas with greater socioeconomic disadvantage, measured using the Socioeconomic Indices for Areas Index of Relative Socioeconomic Disadvantage, the incidence of ICU admission was 50% higher comparing most deprived vs least deprived quintiles.^19^ In the UK, between 2008 and 2021, areas with the highest quintile of children living in low-income families had higher incidence of PICU admission (170.2 per 100 000 child years compared with 97.4 per 100 000 child years in areas with the lowest quintile).^1^ Our data were consistent with these findings in 2 countries with universal health care, albeit with slightly smaller effect sizes. While health care services are provided free of cost in these health care systems, other structural factors may influence baseline health status and access to care among children living in areas of greater socioeconomic disadvantage.

We found that children living in small and rural areas were at higher risk of critical care admission. While this association was not well described in pediatric critical care, these findings were consistent with known increased risk of mortality among children living in rural and remote areas.^20,21^ Several factors may underpin these observed differences in risk of critical care admission. Children who live in rural areas have higher rates of emergency department use and hospitalization for ambulatory sensitive conditions and reduced access to primary and specialist care.^22,23,24,25^ For example, children with CMC in rural areas are more likely to first receive hospital care in nonpediatric facilities than their urban counterparts.^22,23^

The association between situational vulnerability quintile and incidence of critical care admission was present only for urgent admissions in small population centers and rural areas, and children living in small and rural areas with greater situational vulnerability had the highest risk of urgent critical care admission. To our knowledge, this finding has not been previously described in pediatric critical care. A similar interaction has been described among children hospitalized due to motor vehicle collisions in Canada.^26^ One possible explanation is that in larger population centers, unmeasured systemic and structural factors mitigate the association between area-level socioeconomic disadvantage and rates of urgent critical care admission. Baseline health status may be improved in urban areas due to better proximity and access to preventative health services and early childhood education services, as well as improved food security and housing conditions relative to rural areas.^27,28,29,30^ In the face of acute illness or injury, timely access to transportation and acute care services in medium and large population centers may mitigate the progression to critical illness.

### Strengths and Limitations

Our study is strengthened by using several population-level linked data sources, providing a comprehensive picture of critical care admissions in BC, Canada. This study also has limitations. First, our definition of episodes of critical illness was based on location of care. Incidence estimates and trends presented in our study may underestimate population need for critical care interventions. Patients presenting to hospitals with critical illness may receive critical care interventions in alternate acute care settings (eg, emergency departments, pediatric inpatient wards). In regions further from hospitals with dedicated PICUs, these patients may complete their hospital course in their home region due to resource, transport, or weather constraints and would therefore not be captured in our study cohort. This selection bias differentially impacts children who live further from hospitals with ICUs and may result in underestimation of incidence rates of pediatric critical illness in those regions. Similarly, patients who present to hospital with critical illness but die prior to critical care admission would be missed in our study cohort. To measure and address these potential biases, we will undertake future validation studies to develop and evaluate the performance of alternate health administrative data algorithms for identifying pediatric critical illness. Second, though this study was conducted in a universal health care system, we included only registrants to the provincial health care system (MSP) and therefore cannot extrapolate our findings to children who are not eligible for immediate MSP coverage, including visitors, refugee claimants born outside BC, and newly arrived migrants. Third, we categorized place of residence using postal code information provided in the provincial MSP registry file, which is updated annually. Place of residence may be misclassified if families do not update information after relocating within BC. Fourth, our measures of socioeconomic status are area-based measures and do not apply uniformly to all individuals living in the specific area, creating the potential for ecologic fallacies. Published Canadian data suggest that household socioeconomic status and neighborhood socioeconomic status may each independently influence risk of adverse health outcomes, with joint associations being most pronounced among those with highest individual socioeconomic disadvantage living in the most deprived neighborhoods.^31^ More granular household data on socioeconomic status, and indicators of proximity and access to acute care services would provide further insight into reasons for differences in incidence of critical care admission. Finally, our data lacked information on other health equity stratifiers that may contribute to systemic and structural differences in access to health care services and related health outcomes, including gender, education, racialized groups, and Indigenous identity. Future studies will explore these stratifiers in collaboration with the communities they represent.

## Conclusions

In this retrospective cohort study of 13 990 critical care admissions for children age 0 to 17 years, higher rates of critical care admission were observed in areas of greater socioeconomic deprivation and rural areas and small population centers but with a dose-response association between situational deprivation and incidence of urgent critical care admission in small and rural centers only. Public health strategies to address contributing factors that increase risk of critical illness are needed, particularly in neighborhoods with greater socioeconomic deprivation in rural and small areas.

## Notes

Supplement 1.
eMethods.

eReferences.
**eTable 1.** Twenty-five most common primary *ICD*-codes for admission (from highest to lowest) **eTable 2.** Five most common primary *ICD*-codes for urgent vs elective admissions **eTable 3.** Raw vs age and sex standardized incidence rates by economic region, rurality and situationally vulnerability quintile **eTable 4.** Effect modification in sex specific incidence rates by age group **eTable 5.** Joint effects of situational vulnerability quintile and rurality (small or rural vs medium or large) on incidence of critical care admission stratified by admission type **eTable 6.** Sensitivity analysis restricting to 1 admission per participant **eFigure 1.** Flow chart for cohort creation **eFigure 2.** Proportion of admissions to children with chronic medical conditions over study period **eFigure 3.** Incidence rate ratios for situational vulnerability quintile (vs least deprived) within strata of small or rural and medium or large centers

Supplement 2.
Data Sharing Statement
