# Supplementary material for: Socioeconomic Status, Rurality, and Pediatric Critical Care Admission
Source: JAMA Netw Open. 2026 Mar 26;9(3):e263594. doi: 10.1001/jamanetworkopen.2026.3594 (PMC13022738; doi:10.1001/jamanetworkopen.2026.3594)
Supplement: Supplement 1. — eMethods. eReferences. eTable 1. Twenty-five most common primary ICD-codes for admission (from highest to lowest) eTable 2. Five most common primary ICD-codes for urgent vs elective admissions eTable 3. Raw vs age and sex standardized incidence rates by economic region, rurality and situationally vulnerability quintile eTable 4. Effect modification in sex specific incidence rates by age group eTable 5. Joint effects of situational vulnerability quintile and rurality (small or rural vs medium or large) on incidence of critical care admission stratified by admission type eTable 6. Sensitivity analysis restricting to 1 admission per participant eFigure 1. Flow chart for cohort creation eFigure 2. Proportion of admissions to children with chronic medical conditions over study period eFigure 3. Incidence rate ratios for situational vulnerability quintile (vs least deprived) within strata of small or rural and medium or large centers [file jamanetwopen-e263594-s001.pdf]

Data Sharing Statement

Data
Data available: No

Additional Information
Explanation for why data not available: Data sharing statement: This study used linked retrospective data from Population Data BC. Access to data provided by the Data Stewards is subject to approval but can be requested for research projects through the Data Stewards or their designated service providers. Further information regarding data sets used in this project can be found by visiting the PopData project webpage at: https://my.popdata.bc.ca/project_listings/24-051/.
