# Supplementary material for: Socioeconomic Status, Rurality, and Pediatric Critical Care Admission
Source: JAMA Netw Open. 2026 Mar 26;9(3):e263594. doi: 10.1001/jamanetworkopen.2026.3594 (PMC13022738; doi:10.1001/jamanetworkopen.2026.3594)
Supplement: Supplement 2. — Data Sharing Statement [file jamanetwopen-e263594-s002.pdf]

Supplemental Online Content

Bone JN, Shen Y, Harden S, et al. Socioeconomic status, rurality, and pediatric critical care admission. *JAMA Netw Open*. 2026;9(3):e263594.
doi:10.1001/jamanetworkopen.2026.3594

eMethods.

eReferences.

eTable 1. Twenty-five most common primary ICD-codes for admission (from highest to lowest)

eTable 2. Five most common primary ICD-codes for urgent vs elective admissions

eTable 3. Raw vs age and sex standardized incidence rates by economic region, rurality and situationally vulnerability quintile

eTable 4. Effect modification in sex specific incidence rates by age group

eTable 5. Joint effects of situational vulnerability quintile and rurality (small/rural vs medium/large) on incidence of critical care admission stratified by admission type

eTable 6. Sensitivity analysis restricting to one admission per participant

eFigure 1. Flow chart for cohort creation

eFigure 2. Proportion of admissions to children with chronic medical conditions over study period

eFigure 3. Incidence rate ratios for situational vulnerability quintile (vs least deprived) within strata of small/rural and medium/large centres. Quintile 5 is most deprived

This supplemental material has been provided by the authors to give readers additional information about their work.

© 2026 Bone JN et al. *JAMA Network Open*.

eMethods.

The following sections provide further detail on the background and derivation of variables used in the main text.

Exposures

British Columbia Index of Multiple Deprivation

The BCIMD situational vulnerability domain was developed from 2021 census microdata using a Principal Component Analysis (1). It is reported at the level of the community health service area and consists of the proportion of the population aged 25-64 without a high school diploma, the proportion of homes needing major repairs, the proportion of single parent families, the median income, median dollar value of dwellings, and the proportion of the population that is self-employed. The BCIMD is summarized by quintile with the lowest quintile representing the least deprived. The BCIMD data for community health service areas where >25% of the population identifies as Indigenous have been suppressed, consistent with guidance from the First Nations Health Authority (a total of < 5% of the included admissions in main text).

The Pediatric Clinical Classification System

The Pediatric Clinical Classification System (PECCS-CA) groups ICD-10 CA codes into 834 distinct, clinically meaningful categories, which can be further placed categorized as surgical, medical or surgical and medical. The PECCS-CA is a modification of the original US classification system tailored to the Canadian Health care system (2).

Chronic medical conditions

We defined children living with medical complexity (CMC) using the Feudtner methodology adapted by the Canadian Institute for Health Information (CIHI) (3). We used a five-year look-back from the index hospitalization to identify hospital admissions with relevant ICD-10CA and CCI (procedure) codes. Specifically, for each included admission, we extracted any records in the discharge abstract database within the previous five years (including any that were also included in our cohort, i.e., for repeat admissions) and searched these records for relevant codes. We then categorized CMC into specific CIHI categories. We then determined whether a patient had a single category or multiple. We characterized technology dependence using the procedural codes defined by CIHI CMC methodology (3).

Cohort creation

The primary outcome of pediatric critical illness as a unique hospital record in the Discharge Abstract Database with special care unit (SCU) days in a pediatric intensive care unit, combined

© 2026 Bone JN et al. *JAMA Network Open*.

medical/surgical intensive care unit, coronary care unit, or cardiac intensive care unit. To avoid inclusion of admissions related to the perinatal period we excluded admissions only to neonatal intensive care units (defined as SCU days in NICU Level 1, 2, or 3). We retained cases where a patient was transferred from a NICU to a special care unit in the above list. Individuals admitted only to step-down medical/surgical units (Comb med/surg SCU Days) were excluded as these units do not typically admit critically ill children. We combine presumed records according to the same episode of critical care. To determine this, we used two approaches: 1) when a patient was indicated as transferred to another hospital in the discharge field of the Discharge Abstract Database and 2) when two admissions with SCU days (at separate hospitals) were < 24 hours apart. Finally, we excluded any admissions with PICU days from hospitals without a dedicated PICU. Figure S1 outlines the inclusion/exclusion in further detail.

eReferences:

1. Relova S, Joffres Y, Rasali D, Zhang LR, McKee G, Janjua N. British Columbia’s Index of Multiple Deprivation for Community Health Service Areas. Data 2022;7:24.

2. Gill PJ, Thavam T, Anwar MR, et al. Pediatric Clinical Classification System for use in Canadian inpatient settings. PLoS One 2022;17:e0273580.

3. Canadian Institute for Health Information. Children and Youth With Medical Complexity in Canada — Methodology Notes. Ottawa, ON2020.

© 2026 Bone JN et al. *JAMA Network Open*.

<u>**eTable 1:** Twenty-five most common primary ICD-codes for admission (from highest to lowest).</u>

**Primary CA ICD-10 Code**	**Number (% of admissions)**
Acute bronchiolitis	816 (5.8)
Pneumonia	628 (4.5)
Asthma	452 (3.2)
Diabetic ketoacidosis	436 (3.1)
Other lower respiratory disease	421 (3)
Sleep apnea	360 (2.6)
Intracranial injury	256 (1.8)
Poisoning by other medications and drugs	254 (1.8)
Ventricular septal defect	245 (1.8)
Scoliosis	209 (1.5)
Tetralogy of Fallot	192 (1.4)
Other aftercare	182 (1.3)
Status epilepticus	177 (1.3)
Transposition of great vessels	171 (1.2)
Complications of surgical procedures or medical care	162 (1.2)
Crushing injury or internal injury	156 (1.1)
Septicemia (except in labor)	156 (1.1)
Poisoning by psychotropic agents	152 (1.1)
Seizures w and w/o intractable epilepsy	149 (1.1)
Acute upper respiratory infection	147 (1.1)
Complications due to heart valve prosthesis	145 (1)
Ostium secundum atrial septal defect	144 (1)
Inguinal hernia	141 (1)

© 2026 Bone JN et al. *JAMA Network Open*.

eTable 2: Five most common primary ICD-codes for urgent vs elective admissions.

	Urgent (N = 9431)		Elective (N = 4432)	
**Rank**	**Primary ICD-10 CA Code**	**Number (% of admissions)**	**Primary ICD-10 CA Code**	**Number (% of admissions)**
1	Acute bronchiolitis	798 (8.5)	Sleep apnea	304 (6.9)
2	Pneumonia	614 (6.5)	Ventricular septal defect	230 (5.2)
3	Asthma	440 (4.7)	Scoliosis	207 (4.7)
4	Diabetic ketoacidosis	427 (4.5)	Tetralogy of Fallot	148 (3.3)
5	Other lower respiratory disease	381 (4)	Ostium secundum atrial septal defect	140 (3.2)

eTable 3: Raw vs age and sex standardized incidence rates by economic region, rurality and situationally vulnerability quintile.

	**Incidence rate (per 100,000 person years)**	**Age and sex standardized incidence rate (per 100,000 person years)^**
**Population centre type[a]**		
Large (100,000 or greater inhabitants)	146.4	145.6
Medium (30,000 to 99,999 inhabitants)	147.4	147.5
Small (1000 to 29,999 inhabitants)	210.6	212.3
Rural area (<1000 inhabitants)	199.9	203.3
**Situational vulnerability quintile**		
1 (least deprived)	144.4	147.9
2	143.0	142.9
3	169.7	170.6
4	161.2	159.2
5 (most deprived)	189.6	184.9

^Standardized to the pediatric population of British Columbia

© 2026 Bone JN et al. *JAMA Network Open*.

eTable 4: Effect modification in sex specific incidence rates by age group.

	Incidence rate ratio (95% confidence interval) for males vs females
**Age group**	
0-11 months	1.37 (1.28, 1.46)
1-4 years	1.21 (1.15, 1.32)
5-9 years	1.08 (0.99, 1.17)
10-14 years	0.91 (0.84. 0.99)
15-17 years	0.94 (0.86, 1.03)

eTable 5: Joint effects of situational vulnerability quintile and rurality (small/rural vs medium/large) on incidence of critical care admission stratified by admission type.

Situational vulnerability quintile	Rurality	Urgent admissions		Elective admissions	
		Incidence rate	IRR (95% CI)	Incidence rate	IRR (95% CI)
1	Small/Rural	100.1	1.07 (0.95, 1.20)	55.5	1.19 (1.01, 1.40)
2	Small/Rural	114.9	1.21 (1.10, 1.34)	48.7	1.04 (0.90, 1.21)
3	Small/Rural	132.8	1.41 (1.28, 1.55)	44.3	0.95 (0.81, 1.12)
4	Small/Rural	180.8	1.91 (1.3,7, 2.10)	64.6	1.38 (1.18, 1.60)
5	Small/Rural	237.0	2.50 (2.27, 2.74)	51.4	1.09 (0.92, 1.31)
1	Medium/Large	94.5	Reference	46.6	Reference
2	Medium/Large	82.8	0.89 (0.82, 0.96)	50.7	1.09 (0.98, 1.21)
3	Medium/Large	111.8	1.18 (1.10, 1.28)	53.9	1.16 (1.05, 1.29)
4	Medium/Large	88.0	0.93 (0.86, 1.01)	50.7	1.09 (0.98, 1.21)
5	Medium/Large	95.2	1.01 (0.92, 1.10)	52.0	1.11 (0.98, 1.27)

© 2026 Bone JN et al. *JAMA Network Open*.

eTable 6: Sensitivity analysis restricting to one admission per participant

Situational vulnerability quintile	Rurality	Incidence rate ratio (95% confidence interval)*	
		**Primary analysis**	**Sensitivity analysis**
1	Small/Rural	1.10 (1.00,1.21)	1.20 (1.07, 1.34)
2	Small/Rural	1.16 (1.07, 1.25)	1.24 (1.13, 1.36)
3	Small/Rural	1.25 (1.15, 1.35)	1.36 (1.24, 1.49)
4	Small/Rural	1.73 (1.59, 1.87)	1.76 (1.60, 1.93)
5	Small/Rural	2.02 (1.87, 2.19)	2.27 (2.08, 2.49)
1	Medium/Large	Reference	Reference
2	Medium/Large	0.95 (0.90, 1.01)	0.95 (0.89, 1.02)
3	Medium/Large	1.17 (1.10, 1.24)	1.12 (1.04, 1.20)
4	Medium/Large	0.99 (0.92, 1.05)	0.99 (0.92, 1.06)
5	Medium/Large	1.04 (0.97, 1.12)	0.99 (0.90, 1.08)

*Joint effects vs medium/large centres with lowest deprivation quintile

© 2026 Bone JN et al. *JAMA Network Open*.

eFigure 1: Flow chart for cohort creation

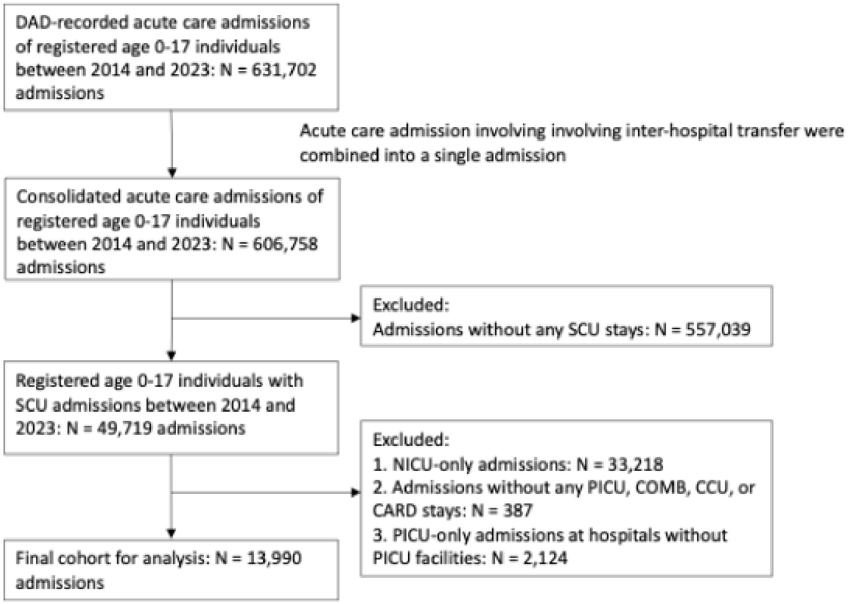

© 2026 Bone JN et al. *JAMA Network Open*.

eFigure 2: Proportion of admissions to children with chronic medical conditions over study period.

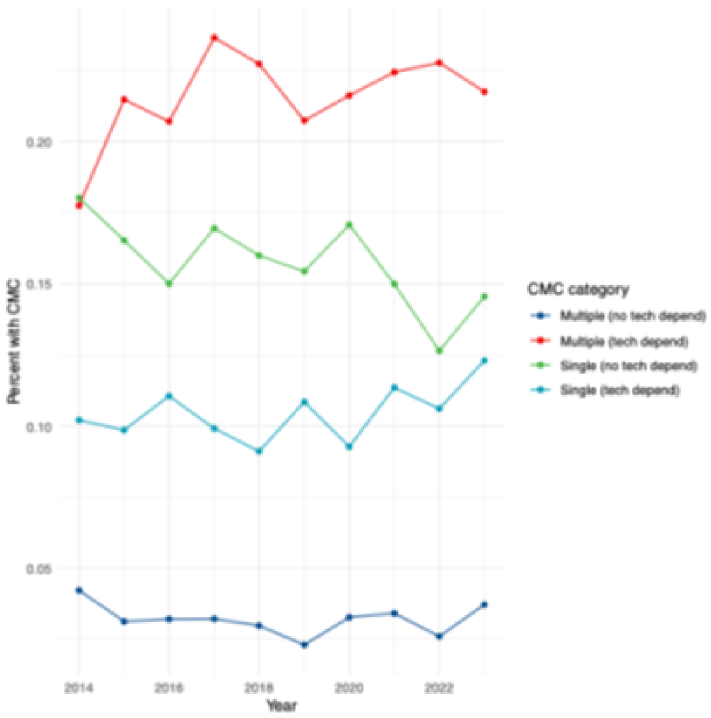

© 2026 Bone JN et al. *JAMA Network Open*.

eFigure 3: Incidence rate ratios for situational vulnerability quintile (vs least deprived) within strata of small/rural and medium/large centres. Quintile 5 is most deprived.

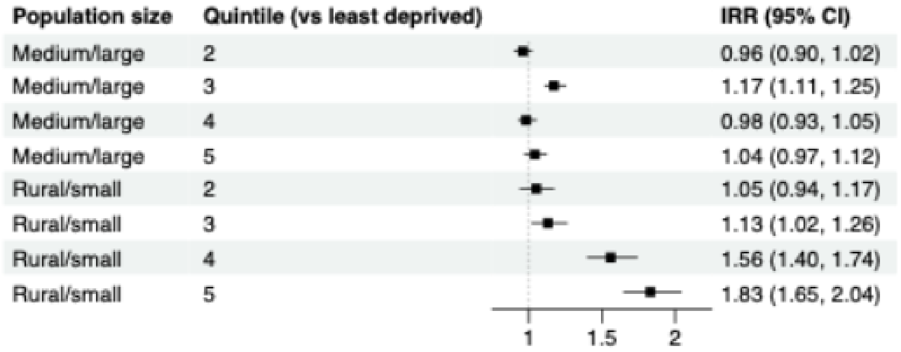

© 2026 Bone JN et al. *JAMA Network Open*.
